# Bystander killing of tumour cells by antibody-targeted enzymatic activation of a glucuronide prodrug

**DOI:** 10.1038/sj.bjc.6690221

**Published:** 1999-03

**Authors:** T L Cheng, S L Wei, B M Chen, J W Chern, M F Wu, P W Liu, S R Roffler

**Affiliations:** Institute of Biomedical Sciences, Academia Sinica, Taipei, Taipei 115, Taiwan; Graduate Institute of Life Sciences, National Defense Medical Center, Taipei, Taipei 100, Taiwan; Department of Biology, Fu Jen Catholic University, Hsin Chuang, Hsin Chuang 242, Taiwan; School of Pharmacy, National Taiwan University, Taipei, Taipei 100, Taiwan; College of Medicine, National Taiwan University, Taipei, Taipei 100, Taiwan

**Keywords:** bystander effect, antibody-directed enzyme prodrug therapy, monoclonal antibody, prodrugs, β-glucuronidase, immunoconjugates

## Abstract

RHI-βG-PEG, formed by linking poly(ethylene glycol)-modified β-glucuronidase to Mab RH1, was employed to examine bystander killing of antigen-negative N1S1 rat hepatoma cells by activation of a glucuronide prodrug (BHAMG) of p-hydroxyaniline mustard (pHAM) at antigen-positive AS-30D rat hepatoma cells. Sequential treatment of cells with 10 μg ml^−1^ RH1-βG-PEG and 20 μM BHAMG was not toxic to N1S1 cells but killed 99% of AS-30D cells. Over 98% of N1S1 cells, however, were killed in mixed populations containing as few as 2% AS-30D cells after identical treatment, demonstrating an in vitro bystander effect. Subcutaneous injection of AS-30D and N1S1 cells in BALB/c *nu/nu* mice produced solid tumours containing both cells. Uptake of radiolabelled RH1-βG-PEG in solid AS-30D and mixed AS-30D/N1S1 tumours was 11.6 and 9.3 times greater than a control antibody conjugate 120 h after i.v. injection. Intravenous treatment with RH1-βG-PEG and BHAMG cured seven of seven nude mice bearing solid s.c. AS-30D tumours and significantly delayed, compared with control conjugate and prodrug treatment, the growth of mixed N1S1/AS-30D tumours with one cure, showing that targeted activation of BHAMG kills bystander tumour cells in vivo. © 1999 Cancer Research Campaign


*British Journal of Cancer* (1999) **79**(9/10), 1378–1385
© 1999 Cancer Research Campaign
Article no. bjoc.1998.0221

### Bystander killing of tumour cells by antibody-targeted enzymatic activation of a glucuronide prodrug

**TL Cheng[1,2], SL Wei[3], BM Chen[1], JW Chern[4], MF Wu[5], PW Liu[3] and SR Roffler[1]**

[1]Institute of Biomedical Sciences, Academia Sinica, Taipei 115, Taiwan; [2]Graduate Institute of Life Sciences, National Defense Medical Center, Taipei 100, Taiwan; [3]Department of Biology, Fu Jen Catholic University, Hsin Chuang 242, Taiwan; [4]School of Pharmacy, National Taiwan University, Taipei 100, Taiwan, and [5]College of Medicine, National Taiwan University, Taipei 100, Taiwan

**Summary** RHI-βG-PEG, formed by linking poly(ethylene glycol)-modified β-glucuronidase to Mab RH1, was employed to examine bystander killing of antigen-negative N1S1 rat hepatoma cells by activation of a glucuronide prodrug (BHAMG) of p-hydroxyaniline mustard (pHAM) at antigen-positive AS-30D rat hepatoma cells. Sequential treatment of cells with 10 μg $ml^{-1}$ RH1-βG-PEG and 20 μM BHAMG was not toxic to N1S1 cells but killed 99% of AS-30D cells. Over 98% of N1S1 cells, however, were killed in mixed populations containing as few as 2% AS-30D cells after identical treatment, demonstrating an in vitro bystander effect. Subcutaneous injection of AS-30D and N1S1 cells in BALB/c *nu/nu* mice produced solid tumours containing both cells. Uptake of radiolabelled RH1-βG-PEG in solid AS-30D and mixed AS-30D/N1S1 tumours was 11.6 and 9.3 times greater than a control antibody conjugate 120 h after i.v. injection. Intravenous treatment with RH1-βG-PEG and BHAMG cured seven of seven nude mice bearing solid s.c. AS-30D tumours and significantly delayed, compared with control conjugate and prodrug treatment, the growth of mixed N1S1/AS-30D tumours with one cure, showing that targeted activation of BHAMG kills bystander tumour cells in vivo.

**Keywords:** bystander effect; antibody-directed enzyme prodrug therapy; monoclonal antibody; prodrugs; β-glucuronidase; immunoconjugates

Monoclonal antibodies have been employed to target therapeutic agents to tumour cells to increase treatment specificity. The therapeutic efficacy of immunoconjugates, however, is limited by their low uptake (Jain, 1990) and heterogeneous distribution in solid tumours (Sung et al, 1993). Antigen-negative or inaccessible tumour cells may escape destruction and repopulate the tumour, leading to treatment failure.

Many of the limitations associated with conventional immunoconjugates may be minimized by antibody-directed enzyme prodrug therapy (ADEPT), in which prodrugs are enzymatically converted to antineoplastic agents by enzyme-immunoconjugates localized at tumour cells (Bagshawe et al, 1988; Senter et al, 1988). Large amounts of antibody-enzyme conjugate can be administered to maximize tumour uptake as the conjugate is nontoxic. In addition, an antineoplastic drug is catalytically produced from a prodrug, allowing accumulation of high drug concentrations at the tumour site (Wallace et al, 1994) and eliminating the need to link drugs directly to antibodies which can damage immunoreactivity (Chari et al, 1995). Antineoplastic drugs generated at antigen-positive tumour cells may also produce a bystander effect by diffusing to neighbouring antigen-negative tumour cells as well as to tumour cells that are inaccessible to antibody conjugates.

Although a bystander effect is expected to be an important advantage of ADEPT, only in vitro evidence of bystander killing by antibody-targeted prodrug activation has been reported (Sahin et al, 1990; Ejigu Retta et al, 1996). We therefore set out to determine if the targeted activation of a glucuronide prodrug (BHAMG) of p-hydroxyaniline mustard (pHAM) could kill antigen-negative bystander tumour cells both in vitro and in vivo. The rat hepatoma cell lines AS-30D and N1S1 were employed in the current study as antigen-positive and antigen-negative tumour cells, respectively. βG was linked to Mab RH1 to target this enzyme to AS-30D cells (Roffler et al, 1994). BHAMG can be preferentially converted to pHAM at AS-30D cells targeted with RH1-βG (Wang et al, 1992). Targeted activation of BHAMG with RH1-βG also displayed potent antitumour activity against malignant ascites in a rat model (Chen et al, 1997) and against solid rat hepatoma tumours in scid mice (Cheng et al, 1997). We now report that the targeted activation of BHAMG produces a bystander effect both in vitro and in vivo.

### MATERIALS AND METHODS

The synthesis of pHAM and BHAMG has been described (Roffler et al, 1991). Bolton-Hunter reagent ($^{125}$I) was purchased from Amersham (Buckinghamshire, UK). Succinimidyl succinate poly(ethylene glycol) MW = 5000 and Sepharose CL-4B protein A were purchased from Sigma Chemical Company, St. Louis, MO, USA. Recombinant βG was produced as described (Cheng et al, 1997).

### Cell lines

The AS-30D rat hepatoma cell line (Smith et al, 1970) was provided by Dr JP Chang, Institute of Zoology, Academia Sinica,

*Received 8 July 1998*
*Revised 1 September 1998*
*Accepted 25 September 1998*

*Correspondence to:* SR Roffler

Taipei. N1S1 rat hepatoma cells were obtained from the American Type Culture Collection (Manassas, VA). Cells were passaged as ascites tumours in Sprague–Dawley rats and cultured in Dulbecco's modified Eagle's medium (Gibco BRL, Grand Island, NY, USA) supplemented with 5% (AS-30D) or 10% (N1S1) heat-inactivated bovine serum, 100 U $ml^{-1}$ penicillin and 100 μg $ml^{-1}$ Streptomycin.

### Antibodies

Mab RH1, an $IgG_{2a}$ monoclonal antibody specific for AS-30D rat hepatoma cells, has been described (Roffler et al, 1994). Hybridoma H16-L10-4R, which secretes a murine $IgG_{2a}$ antibody (Mab HB65) against the nucleoprotein of influenza type A viruses, was obtained from the American Type Culture Collection. Mabs were purified by protein-A affinity chromatography from ascites produced in BALB/c mice. FITC and horseradish peroxidase-conjugated antibodies were from Organon Teknika (Turnhout, Belgium) or Dako (Kyoto, Japan).

### Animals

BALB/c mice were obtained from the animal room of the Institute of Biomedical Sciences, Academia Sinica. BALB/c *nu*/*nu* mice were from the National Laboratory Animal Breeding and Research Center, National Science Council, Taipei. Animal experiments were performed in accordance with institute guidelines.

### Antibody–enzyme conjugates

Recombinant βG derived from *Escherichia coli* was modified with an average of three molecules of methoxy polyethylene glycol (5000 kDa) as described (Cheng et al, 1997) to decrease non-specific binding and extend the serum half-life of the conjugates. PEG modification can also decrease the antigenicity and immunogenicity of βG (manuscript in preparation). PEG-modified βG was linked to Mab RH1 and Mab HB65 by a thioether linkage (Wang *et al*, 1992) to produce the conjugates RH1-βG-PEG and HB65-βG-PEG. The antigen-binding and enzymatic activities of these conjugates have been reported (Cheng *et al*, 1997).

### Membrane immunofluorescence

AS-30D and N1S1 cells were incubated for 1 h at 4°C in 500 μL PBS containing 67 nM Mab or conjugate. The cells were washed twice, incubated 1 h on ice with FITC conjugated goat anti-mouse IgG (Organon Teknika, 1:500) and washed again before the surface immunofluorescence of 10 000 cells was measured with a FACSCalibur flow cytometer (Becton Dickinson, Mountain View, CA, USA). Mean fluorescent intensities were estimated with the Cell Quest software (Becton Dickinson). Background fluorescence was determined by omitting the first antibody before incubation with FITC conjugate.

### In vitro cytotoxicity

AS-30D or N1S1 cells ($10^6$) were suspended in 500 μl of medium containing various concentrations of pHAM or BHAMG for 2 h at 37°C. Cells were then transferred to 6-well plates and cultured in 5 ml medium for 4 days before viable cell numbers in each well were determined by trypan blue exclusion. DMSO, used to dissolve pHAM, was not toxic to AS-30D and N1S1 cells at the maximum final concentrations employed (0.125%). Experiments were carried out in quadruplicate.

### In vitro bystander assay

AS-30D, N1S1 or mixtures of AS-30D and N1S1 cells were suspended in 500 μl of medium. RH1-βG-PEG (10 μg $ml^{-1}$ final concentration) or PBS were added for 30 min before washing the cells three times with PBS. Cells were suspended in 500 μl of medium or medium containing 20 μM BHAMG or 20 μM pHAM for 2 h at 37°C. Cells were then quantitatively transferred to 6-well culture plates in 5 ml fresh medium and cultured for 4 days before viable cell numbers were determined by trypan blue exclusion. Cultures containing both AS-30D and N1S1 cells were incubated with 20 μg $ml^{-1}$ Mab RH1 at 4°C for 1 h, washed twice with PBS, and suspended in PBS containing FITC conjugated goat anti-mouse IgG (1:500) for 30 min. Cells were washed once with PBS before examination of four to 10 fields under visible light as well as under UV illumination with a fluorescent filter to differentiate AS-30D and N1S1 cells. The ratio of AS-30D to N1S1 cells along with the total number of viable cells determined by trypan blue exclusion were used to calculate the number of each cell type present in mixed cultures. Results represent the mean values of four determinations.

### Immunohistochemical staining

Mice were s.c. injected in the right flank with AS-30D cells, N1S1 cells, or mixed AS-30D/N1S1 cells. Tumours were excised after they grew to 200–300 $mm^3$, embedded in O.C.T. compound (Miles Inc., Elkhart, NJ, USA) and snap frozen. Thin sections were incubated with 3 μg $ml^{-1}$ Mab RH1 or Mab HB65 in PBS at 4°C overnight and then sequentially incubated with biotin-labelled rabbit anti-mouse Ig (Dako) and streptavidin-conjugated horseradish peroxidase (Dako) diluted 1:500 in PBS. Binding was visualized with 3,3′-diaminobenzidine before viewing under a light microscope.

### Radiolabelling of conjugates

RH1-βG-PEG and HB65-βG-PEG were labelled with $^{125}I$ Bolton–Hunter reagent to specific activities of 0.73 and 0.72 μCi $μg^{-1}$, respectively, according to the manufacturer's instructions. Conjugates retained antigen-binding activity and specificity as determined by radioimmunoassay against AS-30D and N1S1 cells coated in 96-well microtitre plates. βG activity was unaffected and conjugates were not degraded as determined by autoradiography of gels after sodium dodecyl sulphate polyacrylamide electrophoresis.

### Tumour localization of radiolabelled conjugates

Eighteen BALB/c *nu*/*nu* mice were injected s.c. with $5 \times 10^6$ AS-30D cells in the left flank, $7.5 \times 10^6$ N1S1 cells in the right flank and $10^7$ mixed AS-30D/N1S1 (1:1) cells in the right shoulder. When tumours grew to between 50 and 200 $mm^3$, mice were i.v. injected with 200 μg [$^{125}$I]RH1-BG-PEG or [$^{125}$I]HB65-βG-PEG. Groups of three mice were killed and dissected after 72, 96 and

120 h. Tumours, blood and organs were weighed on an analytical balance and assayed for radioactivity in a multichannel γ-counter. Results are expressed as specific uptake of conjugate in tumour or tissue (cpm $mg^{-1}$) and as localization index, defined as the ratio of specific (RH1-βG-PEG) to nonspecific (HB65-βG-PEG) conjugate uptake in tumour or tissue, normalized to the radioactivity in the blood of mice. Localization index (LI) was calculated by:

$$\text{Localization index} = \frac{(\text{cpm mg}^{-1}\text{ tissue/cpm mg}^{-1}\text{ blood})\text{ RH1-}\beta\text{G-PEG}}{(\text{cpm mg}^{-1}\text{ tissue/cpm mg}^{-1}\text{ blood})\text{ HB65-}\beta\text{G-PEG.}}$$

### Toxicity of prodrug activation by circulating conjugate

Twenty BALB/c mice were i.v. injected with 300 μg RH1-βG-PEG on day 1. Groups of 5 mice were then i.v. injected with 15 mg $kg^{-1}$ BHAMG 72, 96, 120 or 144 h later. The number of white blood cells, red blood cells, and platelets in blood samples collected from the lateral tail vein of mice 1 day before and 9 days after BHAMG treatment were determined as described (Cheng et al, 1997).

### In vivo bystander killing

Groups of 14 BALB/c *nu*/*nu* mice were s.c. injected in the right flank with $3 \times 10^6$ AS-30D cells, $3 \times 10^6$ N1S1 cells or a mixture of $1.5 \times 10^6$ AS-30D and $1.5 \times 10^6$ N1S1 cells on day 1. On day 4, the mice in each group were i.v. injected with either 300 μg RH1-βG-PEG or HB65-βG-PEG followed 5 days later by two i.v. injections (3 h delay) of 15 mg $kg^{-1}$ BHAMG dissolved in PBS. Mice were administered a second round of therapy on day 16 consisting of 200 μg conjugate followed 5 days later with 2 doses of 10 mg $kg^{-1}$ BHAMG. Mouse weight and tumour dimensions were measured biweekly. Tumour volumes were estimated by 0.5 × (length × height × width).

### Statistical analysis

Statistical significance of differences between mean values was estimated with the shareware program Schoolstat (White Ant Occasional Publishing, West Melbourne, Australia) using the independent *t*-test for unequal variances.

### RESULTS

### Characterization of the bystander model

AS-30D rat hepatoma cells were used as antigen-positive tumour cells to examine whether the targeted activation of BHAMG could generate a bystander effect. These cells express a 32 kDa surface antigen that can be targeted with Mab RH1 (Roffler et al, 1994). N1S1 rat hepatoma cells, employed as antigen-negative cells, grew in vitro with a doubling time of 15 h. AS-30D cells grew slightly slower with a doubling time of 19.4 h. Mab RH1 and control Mab HB65 were linked to βG that had been modified with an average of three molecules of methoxy poly(ethylene glycol) to form the conjugates RH1-βG-PEG and HB65-βG-PEG. Table 1 summarizes the binding characteristics of the antibodies and conjugates to AS-30D and N1S1 cells as determined by indirect immunofluorescence and flow cytometric analysis. Mab RH1 and RH1-βG-PEG preferentially bound to AS-30D cells; about 110 times more RH1 and 145 times more RH1-βG-PEG bound to AS-30D cells than to N1S1 cells. RH1-βG-PEG did not specifically bind N1S1 cells as shown by the similar mean fluorescence values of RH1-βG-PEG and HB65-βG-PEG for these cells. N1S1 tumour cells are thus considered to be ‘antigen-negative’ in the present study. The control antibody conjugate, HB65-βG-PEG, did not specifically bind to AS-30D or N1S1 cells (Table 1).

BHAMG was relatively nontoxic to both AS-30D and N1S1 cells with $IC_{50}$ values of 130 and > 200 μM, respectively (Figure 1). pHAM, on the other hand, was over 250 times more toxic with $IC_{50}$ values of 0.2 and 0.8 μM for AS-30D and N1S1 cells, respectively. AS-30D cells were more sensitive than N1S1 cells to pHAM at low concentrations (< 2 μM) but slightly less sensitive at higher drug concentrations.

### In vitro bystander killing

Bystander killing was first examined in cultures containing equal numbers of AS-30D and N1S1 cells. Figure 2 shows that addition of 20 μM pHAM to AS-30D or N1S1 cells resulted in over 99% cell killing. Over 98% of N1S1 and AS-30D cells in a mixed cell culture were also killed by 20 μM pHAM. BHAMG (20 μM), on the other hand, was non toxic to both AS-30D and N1S1 cells, regardless of whether the cells were cultured separately or together. Incubation of AS-30D cells with RH1-βG-PEG followed by exposure to BHAMG resulted in the killing of over 99% of

**Table 1** Mab and conjugate specificity. AS-30D and N1S1 cells were incubated with Mab RH1, Mab HB65, RH1-βG-PEG, or HB65-βG-PEG and FITC conjugated goat anti-mouse IgG before $1 \times 10^4$ cells were analysed for membrane immunofluorescence on a Becton Dickinson FACStar

| Antibody or conjugate | AS-30D cell fluorescent index[a] | N1S1 cell fluorescent index | AS-30D/N1S1 fluorescent ratio[b] | AS-30D cell specific index[c] | N1S1 cell specific index |
|---|---|---|---|---|---|
| Mab RH1 | 1320 | 12.0 | 110 | 185 | 4.8 |
| RH1-βG-PEG | 540 | 3.7 | 146 | 225 | 1.4 |
| Mab HB65 | 7.2 | 2.5 | 2.8 | 1.0 | 1.0 |
| HB65-βG-PEG | 2.4 | 2.7 | 0.9 | 1.0 | 1.0 |

[a]The fluorescent index is the ratio of the mean fluorescence of a Mab or conjugate to the mean background fluorescence (no first antibody or conjugate). [b]The fluorescent ratio is the ratio of the AS-30D to N1S1 fluorescent index for a particular antibody or conjugate. [c]The specific index is the ratio of the fluorescent index of Mab RH1 or RH1-βG-PEG to the fluorescent index of Mab HB65 or HB65-βG-PEG, respectively.

 *© Cancer Research Campaign 1999*

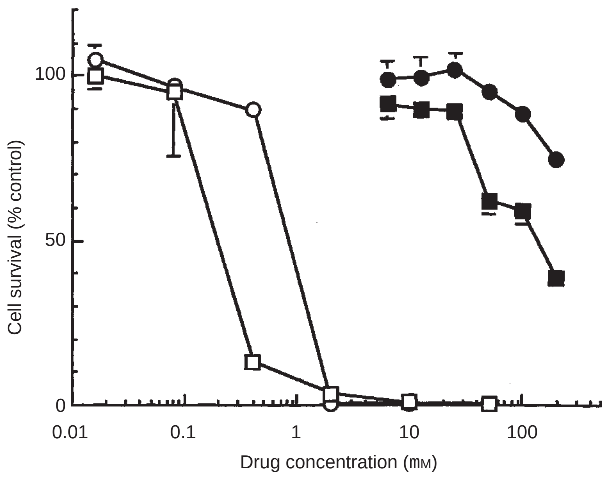

**Figure 1** Sensitivity of tumour cells to pHAM and BHAMG. The viability of AS-30D (squares) or N1S1 (circles) cells were determined 4 days after the cells were exposed to pHAM (open symbols) or BHAMG (solid symbols) for 2 h at 37°C. Results represents the mean viability of cells ($n$ = 4) compared with untreated cells, bars = SE

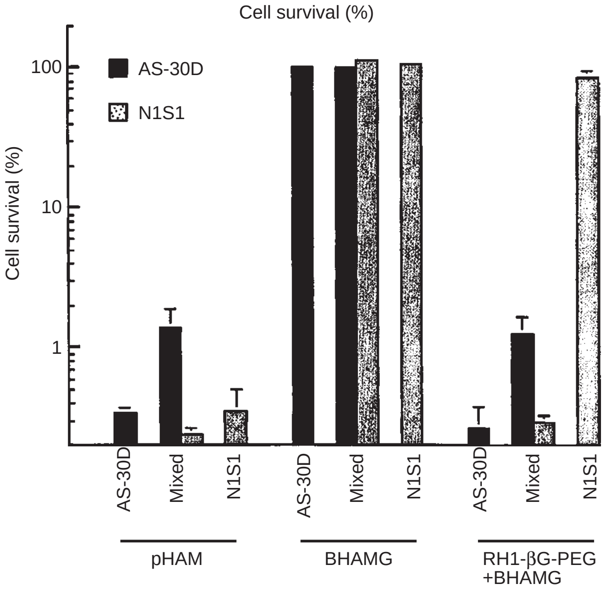

**Figure 2** In vitro bystander killing of antigen-negative cells. Separate or mixed groups containing equal numbers of AS-30D and N1S1 cells were incubated with RH1-βG-PEG or culture medium for 30 min. After washing, cells treated with RH1-βG-PEG were exposed to BHAMG whereas the other groups were treated with pHAM, BHAMG, or medium alone for 2 h. Results show the mean survival of treated cells relative to untreated cells determined 4 days later, bars = SE

these $Ag^+$ cells. N1S1 cells treated in an identical fashion were resistant to the glucuronide prodrug, indicating that these cells were unable to bind RH1-βG-PEG and activate BHAMG. Incubation of a mixture of AS-30D and N1S1 cells with RH1-βG-PEG, in contrast, produced a strong bystander effect upon addition of BHAMG with over 98% killing of both $Ag^+$ and $Ag^-$ tumour cells.

The potency of the bystander effect was examined by performing mixing experiments with different ratios of AS-30D and N1S1 cells. Figure 3 shows that a strong bystander effect was observed after sequential treatment of cells with RH1-βG-PEG and BHAMG in cell populations containing as few as 2% AS-30D cells. The slightly greater killing of N1S1 cells compared with AS-30D cells in mixed cultures can be attributed to the greater sensitivity of N1S1 cells to pHAM concentrations greater than 2 µM.

### Immunohistochemical staining of tumour xenografts

Athymic mice were employed for animal studies to minimize the possibility of immune-mediated bystander killing of tumour cells because we have shown that rats cured of hepatoma ascites by combined treatment with RH1-βG and BHAMG were completely protected from a lethal challenge of AS-30D cells (Chen et al, 1997). The ability of mixed AS-30D and N1S1 tumour xenografts to grow in BALB/c *nu*/*nu* mice was first assessed. Immunohistochemical staining of tumour sections showed that Mab RH1 uniformly stained AS-30D tumours (Figure 4A) but did not bind to N1S1 tumour sections (Figure 4B). Mixed AS-30D/N1S1 tumours exhibited areas of strong staining with Mab RH1 (Figure 4C), demonstrating the presence of AS-30D cells in mixed tumours. Unstained tumour cells were evident after light haematoxylin staining (Figure 4D), indicating that mixed tumours also contained N1S1 cells. AS-30D and N1S1 cells in mixed tumours were nonrandomly distributed with some segregation of the two cell lines (Figure 4D). Mab HB65 did not stain either AS-30D or N1S1 cells (data not shown). AS-30D, N1S1 and mixed AS-30D/N1S1 tumours grew at equivalent rates until tumours reached approximately 200 $mm^3$ at which time N1S1 tumours grew about 25% slower (data not shown).

### Tumour localization of RH1-βG-PEG

The distribution of radiolabelled conjugates in tumour-bearing mice was examined to determine whether RH1-βG-PEG specifically localized in mixed tumours. AS-30D tumours accumulated higher levels of radioactivity compared with normal tissues after i.v. injection of RH1-βG-PEG at all times examined (Figure 5A). RH1-βG-PEG cleared from blood and normal tissues faster than from AS-30D tumours, resulting in progressively higher tumour/tissue ratios from 72 to 120 h. For example, AS-30D/tissue ratios at 120 h ranged from 5.0 ± 0.4 for blood to 28 ± 4.7 for the intestine. Figure 5A shows that RH1-βG-PEG also localized in mixed AS-30D/N1S1 tumours, although specific uptake was about 25% lower than in AS-30D tumours after 120 h (432 vs 318 cpm $mg^{-1}$). HB65-βG-PEG did not localize in any of the tumours (Figure 5B). Calculation of the localization index (Table 2), the ratio of RH1-βG-PEG to HB65-βG-PEG uptake normalized for the serum concentration of the conjugates, indicated selective uptake of RH1-βG-PEG by AS-30D and mixed AS-30D/N1S1 tumours; there was 11.6 times more RH1-βG-PEG than HB65-βG-PEG in AS-30D tumours 120 h after conjugate administration, whereas mixed AS-30D/N1S1 tumours bound over nine times more specific conjugate than HB65-βG-PEG after 120 h. In contrast, the localization index of most tissues was near unity, indicating equal uptake of specific and control conjugates.

© Cancer Research Campaign 1999

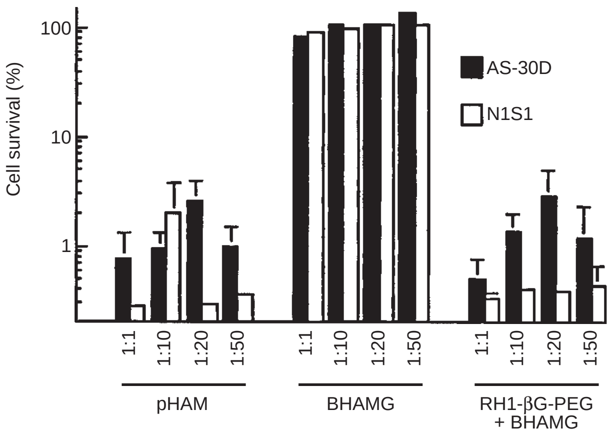

**Figure 3** Potency of in vitro bystander killing. Mixed populations containing different ratios of cells (AS-30D:N1S1) were treated as described in Figure 2. Results show the mean survival of treated cells relative to untreated cells determined 4 days later, bars = SE

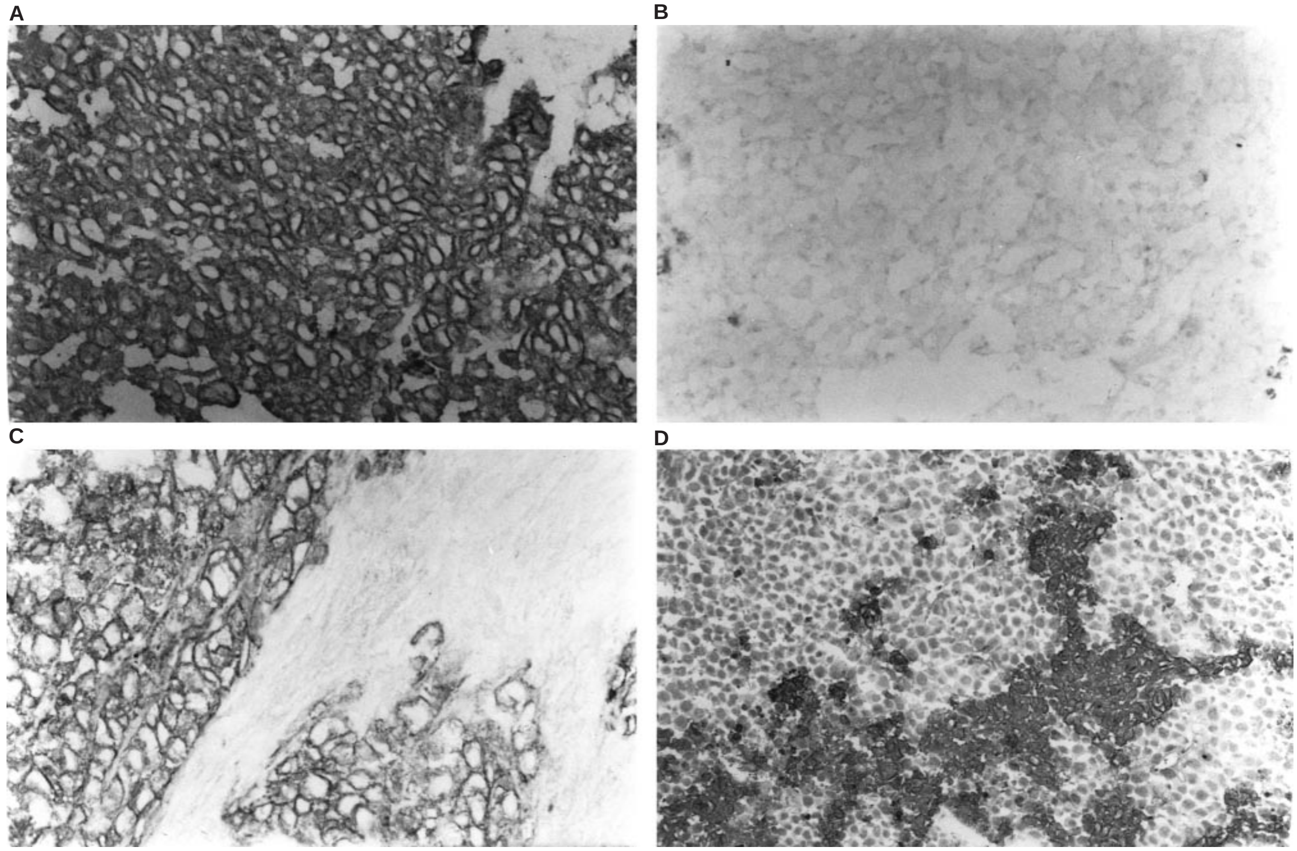

**Figure 4** Immunohistochemical staining of tumour xenografts. Thin sections of AS-30D (**A**), N1S1 (**B**) and mixed AS-30D/N1S1 (**C, D**) tumours were incubated with Mab RH1. Tissue sections were subsequently incubated with biotin-labelled goat anti-mouse Ig and streptavidin-conjugated horseradish peroxidase before addition of substrate. Magnification: **A**, × 200; **B** × 400; **C** × 400; **D**, × 100. The section in panel **D** was counterstained with haematoxylin

The N1S1 tumour localization index was near unity after 72 and 96 h, showing that RH1-βG-PEG did not specifically accumulate in N1S1 tumours at these times. The N1S1 index of 2 at 120 h can be attributed to higher serum concentrations of HB65-βG-PEG (Figure 5B) relative to RH1-βG-PEG (Figure 5A). The N1S1 localization index was not significantly higher than any normal tissue except for the intestine after 120 h.

### In vivo bystander effect

Conversion of BHAMG to pHAM by RH1-βG-PEG present in the circulation could produce systemic toxicity and decrease the therapeutic index of treatment. A single i.v. injection of 15 mg $kg^{-1}$ BHAMG did not decrease white blood cell numbers or animal weight as long as the concentration of RH1-βG-PEG in serum was

 © Cancer Research Campaign 1999

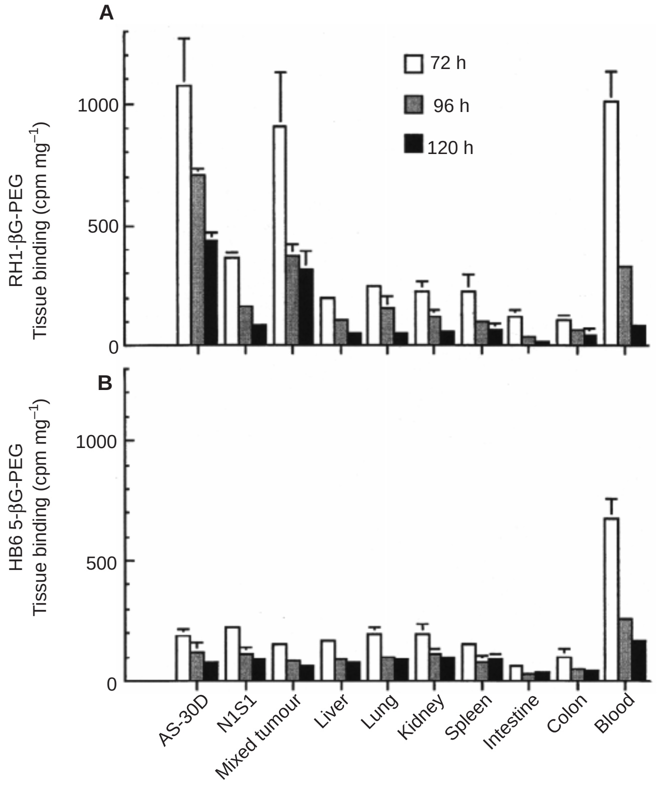

**Figure 5** Biodistribution of [$^{125}$I]labelled conjugates in tumour-bearing BALB/c *nu/nu* mice. Radioactivity of tumours and tissues were determined 72, 96 and 120 h after nude mice bearing AS-30D, N1S1, and mixed AS-30D/N1S1 tumours were i.v. injected with 200 μg RH1-βG-PEG (**A**) or HB65-βG-PEG (**B**). Results represent the mean values of three mice, bars = SE

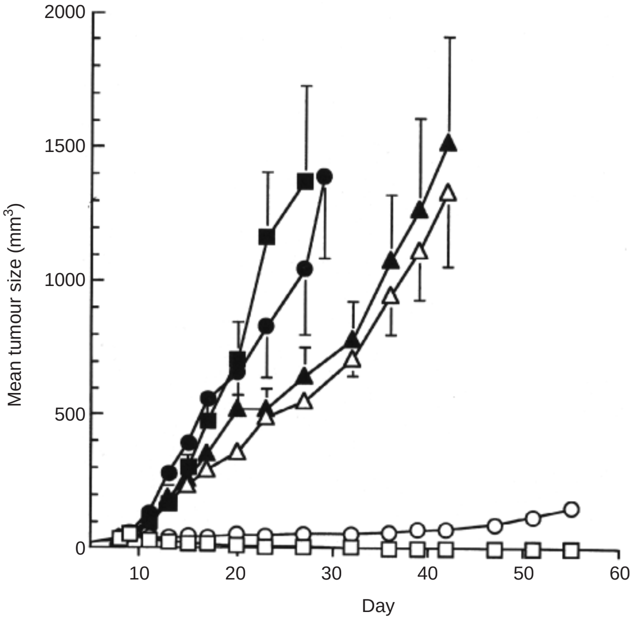

**Figure 6** In vivo bystander effect. Groups of seven BALB/c *nu/nu* mice bearing AS-30D (□), N1S1 (△) or mixed AS-30D/N1S1 (1:1 ratio) (○) tumours were i.v. injected with RH1-βG-PEG (open symbols) or HB65-βG-PEG (solid symbols) on day 4 followed by two i.v. injections of BHAMG on day 9. Mice were again i.v. injected with conjugates on day 16 and BHAMG on day 21. Mean tumour volumes of each group are shown. Bars = SE of the mean. The mean size of AS-30D and mixed AS-30D/N1S1 tumours in mice treated with BHAMG and RH1-βG-PEG were significantly ($P \leq 0.01$) smaller than the corresponding tumours in mice treated with BHAMG and HB65-βG-PEG after day 13

*© Cancer Research Campaign 1999*

**Table 2** In vivo distribution of [$^{125}$I]RH1-βG-PEG compared to [$^{125}$I]HB65-βG-PEG. The mean radioactivity of tissues taken from groups of three athymic mice bearing AS-30D, N1S1 and mixed AS-30D/N1S1 tumour xenografts at the indicated times after i.v. injection of 140 μCi [$^{125}$I]RH1-βG-PEG or [$^{125}$I]HB65-βG-PEG are shown

| | Localization index[a] | | |
|---|---|---|---|
| **Tissue** | **72 h** | **96 h** | **120 h** |
| Blood | 1.00 | 1.00 | 1.00 |
| AS-30D | 3.87 ± 0.39 | 5.55 ± 1.18 | 11.63 ± 1.88 |
| AS-30D/N1S1 | 3.79 ± 0.14 | 3.79 ± 0.90 | 9.26 ± 0.81 |
| N1S1 | 1.11 ± 0.18[b,cc] | 1.22 ± 0.22[b] | 2.05 ± 0.34[b,cc] |
| Liver | 0.80 ± 0.14[b,cc] | 0.95 ± 0.11[b,c] | 1.21 ± 0.13[b,cc] |
| Lung | 0.87 ± 0.05[b,cc] | 1.30 ± 0.43[b,c] | 1.13 ± 0.13[b,cc] |
| Kidney | 0.81 ± 0.08[b,cc] | 0.86 ± 0.11[b,c] | 1.35 ± 0.61[b,cc] |
| Spleen | 1.12 ± 0.55[b,c] | 1.11 ± 0.17[b,c] | 1.25 ± 0.13[b,cc] |
| Intestine | 1.31 ± 0.24[b,cc] | 1.11 ± 0.10[b,c] | 0.85 ± 0.17[b,cc,d] |
| Colon | 0.95 ± 0.47[b,c] | 1.13 ± 0.05[b,c] | 1.82 ± 0.87[b,cc] |

[a]Localization index represents a normalized ratio of RH1-βG-PEG binding compared with HB65-βG-PEG binding in tissues and was calculated as described in Materials and Methods. Results represent the mean ratios ± SE obtained using three mice for each conjugate. Significant differences between AS-30D ([b]$P \leq 0.05$), mixed AS-30D/N1S1 ([c]$P \leq 0.05$; [cc]$P \leq 0.005$) or N1S1 tumour xenografts ([d]$P \leq 0.05$) and other tissues is indicated.

less than about 3 μg ml$^{-1}$. The concentration of RH1-βG-PEG was 7.3 ± 0.7 μg ml$^{-1}$ 4 days and 1.4 ± 0.9 μg ml$^{-1}$ 5 days after i.v. injection of 300 μg ml$^{-1}$ conjugate indicating that a minimum delay of 5 days between RH1-βG-PEG and BHAMG administration would be appropriate for in vivo therapy of solid tumours.

Bystander killing was examined in nude mice which were xenografted with equal numbers of AS-30D and N1S1 cells. Mice were i.v. injected with RH1-βG-PEG or HB65-βG-PEG on day 4 and BHAMG on day 9. All mice received a second round of therapy consisting of i.v. injections of conjugate on day 16 and prodrug on day 21. Figure 6 shows that treatment of mice bearing AS-30D tumours with RH1-βG-PEG and BHAMG resulted in complete tumour regressions in seven of seven mice with all mice appearing tumour-free on day 90. Treatment of mixed AS-30D/N1S1 tumors with RH1-βG-PEG and BHAMG significantly ($P \leq 0.01$ after day 13) suppressed tumour growth compared with mixed tumours which were treated with control conjugate and BHAMG. For example, the mean size of mixed tumours treated with RH1-βG-PEG was 51 mm$^3$ vs 1390 mm$^3$ for mice treated with HB65-βG-PEG on day 29. Furthermore, one of seven mice bearing mixed AS-30D/N1S1 tumours was cured by treatment with RH1-βG-PEG (no evidence of a tumour on day 90). Treatment was specific as shown by the lack of efficacy against N1S1 tumours regardless of whether mice were treated with RH1-βG-PEG or HB65-βG-PEG in combination with BHAMG.

Immunohistochemical staining of frozen sections prepared from mixed tumours treated with RH1-βG-PEG and BHAMG which were recovered on day 55 revealed that viable AS-30D cells were still present (20% of N1S1 cells), indicating selective killing of antigen-positive tumour cells.

### DISCUSSION

The bystander effect produced by the targeted activation of BHAMG was examined in a model employing AS-30D and N1S1 rat hepatoma cell lines. This model was chosen for several reasons: (1) AS-30D cells express a 32 kDa antigen (Roffler et al, 1994) which can be targeted with RH1-βG-PEG, whereas immunofluorescence and histochemical staining of tumours demonstrated that this conjugate did not bind to N1S1 cells; (2) the targeted activation of BHAMG has been shown to exibit antitumour activity against AS-30D tumours. Combined treatment with RH1-βG and BHAMG cured 75% to 100% of rats bearing advanced AS-30D ascites (Chen et al, 1997), while treatment with RH1-βG-PEG and BHAMG cured 100% of mice bearing small solid AS-30D tumours (Cheng et al, 1997). pHAM did not exhibit significant antitumour activity in either model. (3) AS-30D and N1S1 cells displayed similar growth rates and drug sensitivities to pHAM. (4) AS-30D and N1S1 were able to form mixed tumours in nude mice as demonstrated by immunohistochemical staining of tumour sections. Nude mice were employed in this study to eliminate possible immunological-mediated bystander killing of mixed tumours because combined treatment of AS-30D ascites with RH1-βG and BHAMG was shown to induce protective immunity in rats (Chen et al, 1997).

Experiments employing different ratios of AS-30D and N1S1 cells showed that substantial killing of antigen-negative cells was achieved when up to 98% of the cell population contained Ag$^-$ tumour cells. The magnitude of the in vitro bystander effect generated by the targeted activation of BHAMG appears to be at least as great as the bystander effect produced by other enzyme/prodrug combinations. For example, activation of a phosphate prodrug of Mitomycin C by alkaline phosphatase localized at CD30-positive cells by a bispecific antibody produced less cytotoxicity to CD30-negative cells than Mitomycin C in cultures containing equal numbers of Ag$^+$ and Ag$^-$ cells (Sahin et al, 1990). Activation of a phenol mustard prodrug by an antibody-carboxypeptidase G2 conjugate at CEA-positive LS174T colon adenocarcinoma cells produced a strong bystander effect at non CEA-producing lung carcinoma cells in cultures containing equal cell numbers, although the $IC_{50}$ value was about double the value determined for the parent drug (Ejigu Retta et al, 1996). The extent of the bystander effect produced by other enzyme/prodrugs containing different ratios of Ag$^+$ and Ag$^-$ tumour cells has not been reported.

The targeted activation of BHAMG also produced bystander killing of antigen-negative tumour cells in vivo. This was clearly demonstrated by the large difference in the size of mixed AS-30D/N1S1 tumours treated with RH1-βG-PEG or HB65-βG-PEG and BHAMG (51 mm$^3$ vs 1390 mm$^3$, repectively, on day 29). The suppression of mixed AS-30D/N1S1 tumours cannot be ascribed to killing of only the AS-30D cells because the mixed tumours were much smaller than N1S1 tumours treated with RH1-βG-PEG and BHAMG (72 vs 1340 mm$^3$ on day 42). Mixed tumours contained viable AS-30D cells 5 weeks after the last round of prodrug treatment, suggesting that further tumour suppression could be achieved by repeated treatment. A bystander effect was also probably important for the cure of AS-30D tumours, as it is unlikely that RH1-βG-PEG reached every cancer cell given the extremely heterogeneous distribution of RH1-βG in malignant ascites even after direct injection of conjugate into the peritoneal cavity (Chen et al, 1997).

 © Cancer Research Campaign 1999

The bystander effect produced by the targeted activation of BHAMG may help to mitigate the impact of heterogeneous immunoconjugate distribution in tumours. Immunoconjugates are large molecules with molecular weights typically ranging from 60 kDa to more than 200 kDa. Although blood vessels in tumours are 'leaky' (Tomlinson, 1990) and have increased permeability compared with normal tissues (Gerlowski and Jain, 1986), the convection of immunoconjugates into the interstitial space around cancer cells is hindered by the elevated pressures in solid tumours (Less et al, 1992). Antibody uptake therefore ranges from only 0.0006 to 0.02% of the total injected dose per gram of tumour in humans (Douillard et al, 1986; Rosenblum et al, 1991). Immunoconjugate distribution in tumours is also highly heterogeneous due to variable antigen expression and uneven perfusion of blood to different regions of the tumour (Shockley et al, 1992). Immunoconjugate penetration into tumours is also hindered by specific binding of antibody to antigens expressed on tumour cells near blood vessels (Fujimori et al, 1990). In contrast to immunoconjugates, diffusion of low molecular weight drugs is relatively efficient. For example, pHAM is estimated to diffuse over 370 times faster than IgG (Baxter et al, 1992), suggesting that bystander cells may be reached by both convection and diffusion. Our results thus show that the targeted activation of BHAMG produced a bystander effect in vitro and in vivo. This therapeutic strategy may benefit the treatment of tumours that display heterogeneous immunoconjugate uptake.

### ACKNOWLEDGEMENTS

This work was supported by grants from the National Science Council (NSC86-2314-B-001-034) and Academia Sinica, Taiwan.

### REFERENCES

Bagshawe KD, Springer CJ, Searle F, Antoniw P, Sharma SK, Melton RG and Sherwood RF (1988) A cytotoxic agent can be generated selectively at cancer sites. *Br J Cancer* **58**: 700–703

Baxter LT, Yuan F and Jain RK (1992) Pharmacokinetic analysis of the perivascular distribution of bifunctional antibodies and haptens: comparison with experimental data. *Cancer Res* **52**: 5838–5844

Chari RVJ, Jackel KA, Bourret LA, Derr SM, Tadayoni BM, Mattocks KM, Shah SA, Liu C, Blättler WA and Goldmacher VS (1995) Enhancement of the selectivity and antitumor efficacy of a CC-1065 analogue through immunoconjugate formation. *Cancer Res* **55**: 4079–4084

Chen BM, Chan LY, Wang SM, Wu MF, Chern JW and Roffler SR (1997) Cure of malignant ascites and generation of protective immunity by monoclonal antibody targeted activation of a glucuronide prodrug in rats. *Int J Cancer* **73**: 392–402

Cheng TL, Chen BM, Chan LY, Wu PY, Chern JW and Roffler SR (1997) Poly(ethylene glycol) modification of β-glucuronidase-antibody conjugates for solid tumor therapy by targeted activation of glucuronide prodrugs. *Cancer Immun Immunother* **44**: 305–315

Douillard JY, Lehur PA, Aillet G, Kremer M, Bianco-Arco A, Peltier P and Chatal JF (1986) Immunohistochemical antigenic expression and in vivo tumor uptake of monoclonal antibodies with specificity for tumors of the gastrointestinal tract. *Cancer Res* **46**: 4221–4224

Ejigu Retta BM, Burke PJ, Photiou A, Melton R and Eno-Amooquaye E (1996) Antibody-directed enzyme prodrug therapy (ADEPT): evidence for a bystander effect in vitro. *Int J Oncol* **9**: 567–570

Fujimori K, Covell DG, Fletcher JE and Weinstein JN (1990) A modeling analysis of monoclonal antibody percolation through tumors: a binding-site barrier. *J Nucl Med* **31**: 1191–1198

Gerlowski LE and Jain RK (1986) Microvascular permeability of normal and neoplastic tissues. *Microvascular Res* **31**: 288–305

Jain RK (1990) Physiological barriers to delivery of monoclonal antibodies and other macromolecules in tumors. *Cancer Res* **50**: 814–819

Less JR, Posner MC, Boucher Y, Borochovitz D, Wolmark N and Jain RK (1992) Interstitial hypertension in human breast and colorectal tumors. *Cancer Res* **52**: 6371–6374

Roffler SR, Wang SM, Chern JW, Yeh MY and Tung E (1991) Anti-neoplastic glucuronide prodrug treatment of human tumor cells targeted with a monoclonal antibody–enzyme conjugate. *Biochem Pharmacol* **42**: 2062–2065

Roffler SR, Chan J and Yeh MY (1994) Potentiation of radioimmunotherapy by inhibition of topoisomerase I. *Cancer Res* **54**: 1276–1285

Rosenblum MG, Kavanagh JJ, Burke TW, Wharton JT, Cunningham JE, Shanken LJ, Silva EG, Thompson L, Cheung L, Lamki L and Murray JL (1991) Clinical pharmacology, metabolism, and tissue distribution of $^{90}$Y-labeled monoclonal antibody B72.3 after intraperitoneal administration. *J Natl Cancer Inst* **83**: 1629–1636

Sahin U, Hartmann F, Senter P, Pohl C, Engert A, Diehl V and Pfreundschuh M (1990) Specific activation of the prodrug mitomycin phosphate by a bispecific anti-CD30/anti-alkaline phosphatase monoclonal antibody. *Cancer Res* **50**: 6944–6948

Senter PD, Saulnier MG, Schreiber GJ, Hirschberg DL, Brown JP, Hellström I and Hellström KE (1988) Anti-tumor effects of antibody-alkaline phosphatase conjugates in combination with etoposide phosphate. *Proc Natl Acad Sci USA* **85**: 4842–4846

Shockley TR, Lin K, Sung C, Nagy JA, Tompkins RG, Dedrick RL, Dvorak HF and Yarmush ML (1992) A quantitative analysis of tumor specific monoclonal antibody uptake by human melanoma xenografts: effects of antibody immunological properties and tumor antigen expression levels. *Cancer Res* **52**: 357–366

Smith DF, Walborg EF and Chang JP (1970) Establishment of a transplantable ascites variant of a rat hepatoma induced by 3′-methyl-4-dimethylaminoazobenzene. *Cancer Res* **30**: 2306–2309

Sung C, Dedrick RL, Hall WA, Johnson PA and Youle RJ (1993) The spatial distribution of immunotoxins in solid tumors: assessment by quantitative autoradiography. *Cancer Res* **53**: 2092–2099

Tomlinson E (1990) Biological dispersion and the design of site-specific protein therapeutic systems. In *Targeting of Drugs 2, Optimization Strategies*. Gregoriadis G, Allison AC, Poste G (eds), pp 1–19. Plenum Press: New York

Wallace PM, Macmaster JF, Smith VF, Kerr DE, Senter PD and Cosand WL (1994) Intratumoral generation of 5-fluorouracil mediated by an antibody-cytosine deaminase conjugate in combination with 5-fluorocytosine. *Cancer Res* **54**: 2719–2723

Wang SM, Chern JW, Yeh MY, Ng JC, Tung E and Roffler SR (1992) Specific activation of glucuronide prodrugs by antibody-targeted enzyme conjugates for cancer therapy. *Cancer Res* **52**: 4484–4491

*© Cancer Research Campaign 1999*
